# Medical Society of the County of Erie

**Published:** 1909-12

**Authors:** Franklin C. Gram


### SOCIETY PROCEEDINGS.

### Medical Society of the County of Erie

Reported by FRANKLIN C. GRAM, M. D., Secretary.

A REGULAR meeting of the Medical Society of the County of Erie was held in the Y. M. C. A. Building, October 18, 1909.

The meeting was called to order by the president, Dr. Charles A. Wall, at 8.45 p. m.

Dr. Gram, secretary, being absent, Dr. Lytle was appointed secretary protempore.

Dr. T. H. McKee, chairman of the Membership Committee. presented applications for membership as follows: William Ward Plummer, Samuel A. Moore, J. W. Flannery, George G. Wagner, Michael A. Sullivan, Hugh McIntyre, Richard J. Pearson, Julia N. Wood, Alois Jokl, Frederick Zingsheim, Robert F. Sheehan, George W. Wheeler, Frederick Seilheimer, Leo J. Doll, O. S. McKee, Karl F. Eschelman, Francis G. Barnes, E. A. Southall, Almon H. Cooke, Frank B. Seitz, Albert E. Mott, Jesse N. Roe, Lee D. Gunn, Eva V. Mead.

Ballots were cast for the aforementioned candidates who were duly elected to membership, and, on motion, the membership committee was tendered a vote of thanks for efficient work.

On motion, the reading of the minutes of the last regular meeting was dispensed with inasmuch as such minutes had been published in the Buffalo Medical Journal.

The minutes of the meetings of the council. held July 14, September 21, October 4, October 15 and October 18, 1909, were read and, it was moved that they be accepted and made a part of these minutes.

Dr. Grosvenor moved to amend by adding "except that so much of the minutes of the council of October 4 as relates to a special committee on professional relation be laid on the table." Lost.

The original motion was then put and carried.

The following are the amendments contained in the minutes of the council meetings of October 4 and 18, 1909, and adopted by the approval of such minutes at this meeting:

### STANDING RULES OF THE SOCIETY.

1. The president shall appoint a special committee on necrology whose duty it shall be to report, at the annual meeting, upon all members dying during the year.

2. The president may appoint a special committee on professional relations, whose duty it shall be to report to him and act only under his personal supervision.

3. A special committee on program and on entertainment may be chosen by the council, to report to them.

4. The president shall designate a member of the board of censors or of any standing committee, as acting chairman with full power, at any time the duly elected chairman is unable to act.

5. Unless otherwise directed, all reports of officers, censors and standing committees shall be submitted to the council before being presented to the society.

6. A special committee on publicity may be appointed by the president, which shall be under the direction of and shall report to the council.

7. The council shall report at each regular meeting as well as at the annual meeting.

AMENDMENTS TO BY-LAWS.

*Chapter XII.*

Sec. 4. Honorary permanent members shall have regular dues and assessments paid by the society.

*Chapter XIII.*

Sec. 2. Standing rules may be made or changed at any regular meeting by a two-thirds vote, provided twenty members are present.

On motion, it was decided to dispense with nominations of candidates at this meeting.

Dr. John H. Grant, chairman of the Board of Censors, read his report which was ordered received and spread upon the minutes as follows:

REPORT OF THE CENSORS.

The abortion case against Mrs. Bailey of Kenmore, heretofore reported as before the Grand Jury, was heard and the Grand Jury refused to indict, although the evidence seemed conclusive. The woman had previously been indicted for manslaughter in an abortion case; but, through unknown influences, she was never brought to trial.

It is suggested that cases of this kind, relating to persons not physicians, are unsuitable for the board of censors, but should receive the attention of the police and district attorney.

August 5, 1909.—Case of William F. Bell, heretofore reported as arrested for practising medicine without a license, after several adjournments in Police Court was again called and Bell had failed to pass the examination held June 21, 24, 1909, and his counsel asked for more delay. Adjourned to August 7, on which date the case was further adjourned to March 3, 1910, to permit Bell to be reëxamined in two subjects in which he failed, his counsel stipulating that his client will abstain from practising medicine until that time.

August 11, 1909.—Investigated the case of a midwife,—one Mrs. Moritz, 101 Kehr street,—reported to the chairman by the Health Department for attending a woman in miscarriage. The chairman saw the woman,—married, with one child,—who denied having had a miscarriage. As she was hard at work scrubbing and washing dishes in a saloon, there appeared to be no evidence and the case was dropped.

October 8, 1909.—A warrant was sworn out and served on Mrs. Dr. M. E. Lane, 122 Whitney Place, for practising medicine without being lawfully qualified, and not registered. She was arraigned in Police Court, October 9, and after hearing the evidence the court held her for the action of the grand jury.

This woman was doing a "land office" business, with waiting room crowded with women patients of all degrees, some finely and some poorly dressed,—all waiting patiently their turn to see "Dr. Lane." It appears the doctor (?) would not permit would-be patients to tell her of their ailments; she stopped them, saying she was the "diagnoser." She then went into contortions while sitting in a chair and told her patient that a Dr. Springsteen in the "spirit" world would diagnose through her, Lane's body, and outline the treatment. When the contortions were over, Dr. Lane told the patient her trouble and there were many pleura growing into the lungs—a condition she called "contention," whatever that may mean—and gave the patient an ounce bottle of medicine, charging her $1.25.

October 18, 1909, your chairman, spent all the afternoon waiting to go before the grand jury with his witnesses in this case, but was unable to get it before that august body as they promptly adjourned at four o'clock, which means another half-day tomorrow for witnesses and chairman.

The scientific part of the meeting was then carried out as follows: J. F. Fairbairn, a paper on Otitis Media in Infants; Surgical Tuberculosis, by W. W. Plummer; Late Tendencies in the Treatment of Surgical Tuberculosis, by Prescott LeBreton, and Commercialism in Medicine, by J. V. Woodruff.

The papers were splendidly discussed. A collation followed, sixty members being present, after which the meeting adjourned.

